# Reproductive-Toxicity-Related Endpoints in *C. elegans* Are Consistent with Reduced Concern for Dimethylarsinic Acid Exposure Relative to Inorganic Arsenic

**DOI:** 10.3390/jdb11020018

**Published:** 2023-04-26

**Authors:** Jessica A. Camacho, Bonnie Welch, Robert L. Sprando, Piper R. Hunt

**Affiliations:** Office of Applied Research and Safety Assessment, Center for Food Safety and Applied Nutrition, Food and Drug Administration, 8301 Muirkirk Road, Laurel, MD 20708, USA

**Keywords:** reproductive-toxicity-related, arsenic, mercury, organic, inorganic, *C. elegans*, small model organism

## Abstract

Exposures to arsenic and mercury are known to pose significant threats to human health; however, the effects specific to organic vs. inorganic forms are not fully understood. *Caenorhabditis elegans’* (*C. elegans*) transparent cuticle, along with the conservation of key genetic pathways regulating developmental and reproductive toxicology (DART)-related processes such as germ stem cell renewal and differentiation, meiosis, and embryonic tissue differentiation and growth, support this model’s potential to address the need for quicker and more dependable testing methods for DART hazard identification. Organic and inorganic forms of mercury and arsenic had different effects on reproductive-related endpoints in *C. elegans*, with methylmercury (meHgCl) having effects at lower concentrations than mercury chloride (HgCl_2_), and sodium arsenite (NaAsO_2_) having effects at lower concentrations than dimethylarsinic acid (DMA). Progeny to adult ratio changes and germline apoptosis were seen at concentrations that also affected gravid adult gross morphology. For both forms of arsenic tested, germline histone regulation was altered at concentrations below those that affected progeny/adult ratios, while concentrations for these two endpoints were similar for the mercury compounds. These *C. elegans* findings are consistent with corresponding mammalian data, where available, suggesting that small animal model test systems may help to fill critical data gaps by contributing to weight of evidence assessments.

## 1. Introduction

Toxicological testing is critical for assessing the safety of chemicals we encounter daily. The U.S. Food and Drug Administration (FDA) aids in safeguarding our food supply by monitoring a broad range of chemicals [1,2]. Unfortunately, the number of chemicals in need of testing ranges in the thousands [3], making it increasingly difficult to quickly assess all of them. Historically, scientists have relied heavily on mammalian studies to evaluate toxicity, but these studies can be expensive and time consuming, and are increasingly criticized for ethical considerations [4]. For developmental and reproductive toxicology (DART) studies, OECD (Organisation for Economic Co-operation and Development) guidelines describe apical endpoint assessments of pre-, post-, and perinatal development and multiple generation testing in rodents and nonrodent mammals [5,6]. Based on the number of the compounds in need of testing, the timing, number, and availability of animals needed, there is an extensive backlog for further assessment and clinical trials [7,8]. New toxicological tools and approaches that better predict the human response with reduced time and expense will allow for the rapid evaluation of many more individual compounds of concern and may facilitate the increased toxicity testing of mixtures.

Funding for the development of alternative models for DART testing is increasing, along with pressure to reduce, refine, and/or replace testing on mammals [9,10]. Strategic plans are underway at the U.S. FDA, other government agencies, and internationally to support efforts to identify and produce faster, cheaper, and more reliable methods for predictive safety assessment and hazard identification. These efforts include the development and evaluation of new alternative methods (NAMs) of testing strategies using in vitro approaches and/or small model organisms (SMOs) such as *Caenorhabditis elegans* (*C. elegans)*, drosophila, and zebrafish [11,12]. Some advantages of working with SMOs for DART studies is the ability to quickly study the direct effects of chemicals on progeny in the absence of maternal metabolism and toxicity, the vast amount of information available about their embryology, and the opportunity to work with complete embryo models instead of less complex cell culture models [13]. As attention is increasingly being focused on designing integrated approaches to DART testing and assessment using NAMs, SMO-based assays can be used as a complementary component to cell-based assays and existing mammalian DART studies in support of safety assessment, hazard identification, and regulatory decision making [14].

*C. elegans* are relatively inexpensive to study and are not considered as animals according to relevant animal welfare acts and regulations [4]. Due to their transparent cuticle and microscopically visible reproductive system, *C. elegans* are a good alternative model system for studying chemical exposure effects on the germline. Many genes and pathways involved in regulating key DART-related processes such as germ stem cell renewal and differentiation, meiosis, and embryogenesis are conserved between *C. elegans* and humans [15]. Though *C. elegans* cannot model follicle stimulation and function, implantation, or placental transfer, they can model germ cell proliferation and differentiation, the clearance of unfit germ cells through apoptosis, meiosis, the maturation of gametes and fertilization, early embryonic development, growth and organ development (organogenesis), the onset of sexual function, and second-generation reproductive capacity [16], making them a potentially useful model for inclusion in integrated DART-related weight of evidence assessments.

Arsenic and mercury are known to be harmful to babies and children, and yet differences in the toxicities of their organic versus inorganic forms require further investigation for hazard prediction [17,18,19]. The FDA’s Center for Food Safety and Applied Nutrition (CFSAN) has issued two guidance documents that apply specifically to inorganic arsenic (iAs) in foods [20,21], and the Environmental Protection Agency (EPA) and FDA have set limits of allowable mercury species in drinking water and seafood, respectively [22,23]. Organic arsenic (oAs) in the form of dimethylarsinic acid (DMA) has been found in the pups of rodent dams fed iAs [24], yet there is little experimental data available from DART studies on DMA [20]. While mammalian toxicity data for oAs species are limited, available data on organic and inorganic forms indicate that iAs is generally considered to be more toxic than oAs [20]. In the case of mercury, elevated concentrations are associated with numerous reproductive defects in humans and animal models [25]. In studies directly comparing mercury chloride (HgCl2, inorganic mercury) and methyl mercury (meHgCl, organic mercury) in rodents or *C. elegans*, meHgCl is more toxic than HgCl2 during early development, juvenile growth, and for several reproductive endpoints [26,27,28]. Consistent with mammalian data, we previously found that for developmental toxicity and oxidative stress endpoints in *C. elegans*, monomethylation increased mercury toxicity while dimethylation decreased arsenic toxicity [28]. Here, we assessed the effects of DMA, sodium (meta)arsenite (NaAsO_2_), HgCl_2_, and meHgCl on the *C. elegans* reproductive-toxicity-related endpoints of progeny output and germline health, as measured using germline nuclear apoptosis and germline histone regulation. Our work seeks to determine how a response in *C. elegans* can be associated with known effects in standardized models for safety testing so that we may better understand how the model can be utilized in hazard identification and subsequently in the regulatory decision-making process.

## 2. Materials and Methods

### 2.1. Chemicals and Dosing

Test chemicals (Table 1) and assay specific positive controls were purchased from MilliporeSigma (Burlington, MA, USA) and Fisher Scientific (Waltham, MA, USA). Fresh 10X dosing solutions were prepared for each experiment in Milli-Q purified water within three hours of dosing. Test chemicals readily dissolved in water.

### 2.2. Worm Maintenance

*C. elegans* wild-type N2, PD4251 (ccIs4251 I; dpy-20(e1282) IV.), and NL2507 (pkIs1582 [let-858::GFP + rol-6(su1006)]) strains were obtained from the Caenorhabditis Genetics Center (CGC), which is funded by the NIH Office of Research Infrastructure Programs (P40 OD010440). Purchased strains were initially grown for three generations on agar plates under continuously well-fed conditions using OP50 *E. coli* as a feeder organism prior to egg isolation and transfer to *C. elegans* Habitation Medium (CeHM) as previously described [29]. Wildtype N2 *C. elegans* larvae grown in CeHM developed at the same rate as those fed OP50 *E. coli* [30,31], and the additional strains utilized in this study grew at similar rates to the wildtype strain. *C. elegans* strain aliquots were frozen at −80 °C soon after transfer to CeHM, and fresh aliquots were thawed every 6 months to avoid genetic drift in test cohorts. All cultures were fed fresh CeHM at least twice a week in biological safety cabinets using a sterile technique. Unless otherwise indicated, cultures were maintained in vented, polystyrene flasks at 20 °C on shakers in hot/cold incubators. To minimize the effects of freezing–thawing on stress resistance and gene expression, thawed *C. elegans* strains were allowed a minimum of three weeks growth in CeHM prior to toxicity testing. To obtain age-synchronized test cohorts, well-fed gravid *C. elegans* were subjected to hypochlorite treatment to isolate eggs. Eggs were allowed to hatch overnight in non-nutrient M9 buffer for 19 +/− 1 h. Only egg isolates containing <1 dauer per approximately 5000 eggs were utilized to ensure that cohorts originated from well-fed cultures and were exposed to minimal amounts of dauer pheromone. Synchronized first-larval-stage worms (L1s) were centrifuged, resuspended to approximately 1 worm per µL CeHM, and monitored for dosing at the fourth larval (L4) or young adult (yA) stage. 

### 2.3. Progeny Ratio

**Scheme 1:**
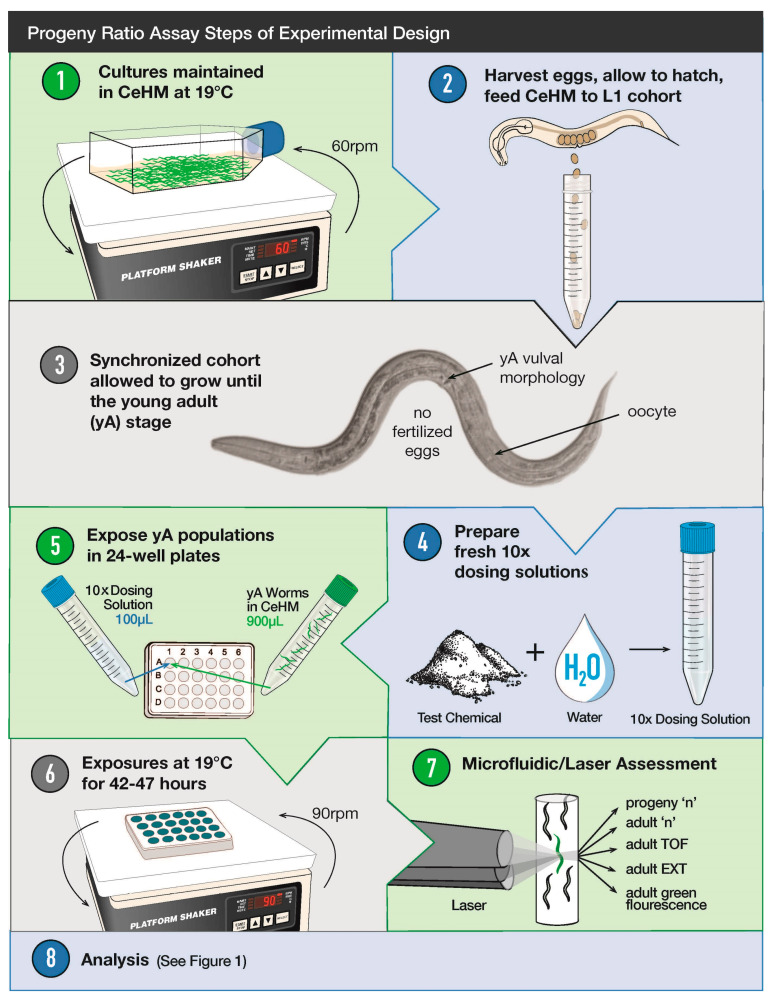
Progeny ratio assay. This assay provides an assessment of the proportion of progeny to adults in the control and exposed populations, as well as adult morphometry.

**Figure 1:**
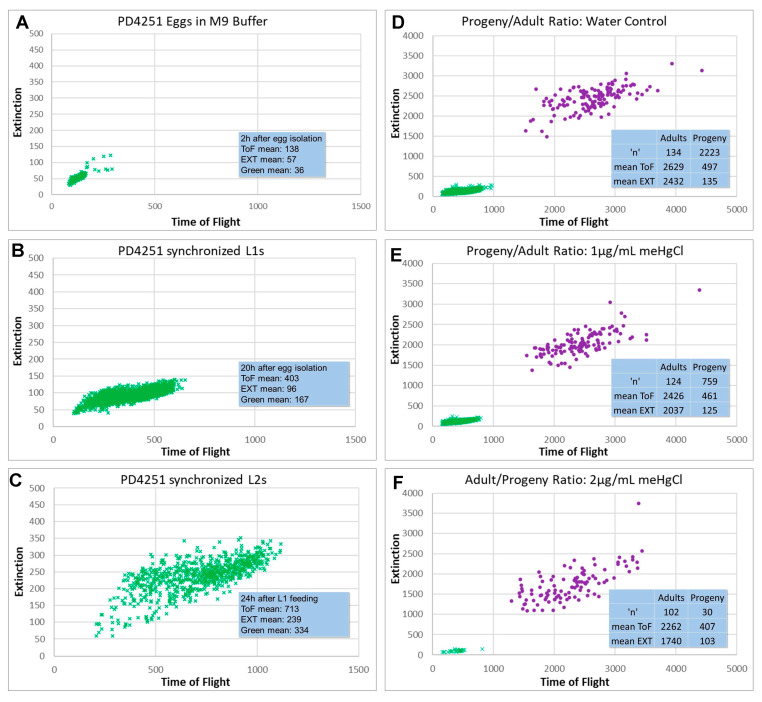
Examples of COPAS data for assessment of progeny ratios and parental gross morphology parameters. (**A**) *C. elegans* PD4251 strain eggs with a few newly hatched L1s two hours after egg isolation. (**B**) L1s allowed to hatch in non-nutrient M9 buffer for 20 h. (**C**) Washed L2s, 24 h after L1 feeding. (**D**–**F**) Parental population (purple dots) and progeny population (green Xs). In these examples from a single experiment, as the concentrations of meHgCl increased, the means for ToF (time of flight, a measure of length) and EXT (extinction, a measure of optical density) decreased slightly for both the parental and progeny populations, while the ratio of progeny to adult ‘n’ decreased dramatically. Note that the x- and y-axes in (**A**–**C**) are up to 1500 for ToF and 500 for EXT, while these values in the progeny ratio examples (**D**–**F**) are 5000 and 4000 for ToF and EXT, respectively. ToF, EXT, and green fluorescence are given in COPAS specific units and ‘n’ is the number of individuals as counted by the COPAS in each population.

This progeny-to-adult ratio assay assesses the number of progeny per adult in a population, and then normalizes that value to the matched water control from the same plate. It also provides information on adult parameters of time-of-flight (ToF, a measure of size), extinction (EXT, a measure of optical density), and green fluorescence. Synchronized PD4251 L1 cohorts were obtained and fed as described above. *C. elegans* maintenance at 19 °C allows all steps of the progeny ratio to be conducted during the daytime, and so a dedicated incubator was used (Scheme 1, Steps 1 and 2). At 19 °C, in CeHM, the PD4251 L4-to-adult molt occurred at around 70–72 h. Age-synchronized yA cohorts were allowed to settle in 15 mL conical tubes and fed fresh CeHM with a dilution of 100–300 worms per mL. A total of 100 µL of water or 10X dosing solutions were added to 900 µL of yA PD4251s in CeHM per well in 24-well plates 1–2 h after the majority of *C. elegans* had passed the final developmental lethargus stage, as assessed visually and using a wMicroTracker^TM^ (wMT, InVivo Biosystems, Eugene, OR, USA) [32] (Scheme 1, Steps 3–5). On the 5th day post-L1 feeding (d5pL1f), plates were analyzed using a Complex Object Parametric Analyzer and Sorter (COPAS^TM^, Union Biometrica, Holliston, MA, USA) with the LP Sampler^TM^ option (Scheme 1, Steps 6 and 7). The use of the PD4251 strain allowed for the removal of noise by selecting for COPAS readings with a green fluorescence value above the background. Progeny populations were then clearly separated from the parental adult population at parental d5pL1f using ToF (time-of-flight, a measure of size) and EXT (extinction, a measure of optical density) (Figure 1). Statistical significance was determined using Student’s *t*-test with a cut off for meaningful biological significance set at 10% due to instrument variability.

### 2.4. Germline Health—Apoptosis

Synchronized N2 L1 cohorts were obtained and fed as described above and maintained for an additional two days until they reached the L4 larval stage (50–52 h post-L1 feeding). Age-synchronized L4 worms were allowed to settle in 15 mL conical tubes, and were then fed with fresh CeHM at a concentration of ~1000 worms per mL. A total of 900 µL of this worm mixture was added to individual wells in 24-well plates with 100 µL of water or a 10X dosing solution of interest, and then it was incubated for 24 h at 20 °C on a shaker at 60 rpm in a hot/cold incubator. After incubation, apoptosis assay was performed via acridine orange staining on synchronized adult N2 hermaphrodites collected 24 h post-exposure (Day 1 adults), as previously described [33,34]. Bisphenol-A (BPA) was used as the positive control [35].

### 2.5. Epigenetic Germline De-Silencing—Histone Regulation Assay

The exposure and GFP germline de-silencing assessments were performed as previously described [36]. Worms were prepared in the same manner as described for the apoptosis assessment, but only with the NL2507 transgenic strain. After incubation, worms were washed 2 times with M9, placed on slides, and scored for germline GFP expression using a Nikon 80i microscope at 20–40× magnification. Worms were scored individually by eye as positive or negative for germline GFP, with representative images captured for confirmation (*n* = 30–35 per condition, in 3–4 replicates). 3-Deazaneplanocin A (DZnep), a histone methyltransferase inhibitor, was used as the positive control [35].

## 3. Results

### 3.1. Progeny/Adult Ratio 

First-larval-stage (L1) progeny can easily be lost during the wash steps; therefore, a method to assess progeny to adult ratios that avoids washing prior to microfluidic/laser evaluation with the COPAS was sought. The *C. elegans* PD4251 strain contains green fluorescent protein (GFP) transgenes encoded with nuclear and mitochondrial protein localization signals [37]. This bright green fluorescing strain was selected for this assay because at 20 °C in CeHM it develops and reproduces at the same rate as wild-type N2 *C. elegans*. 

The mean green fluorescence readings from nutrient media CeHM alone are ~2 COPAS specific units, while in an initial exploration of the PD4251 model for progeny ratio assessment, the mean green COPAS-specific values for PD4251 eggs, L1s, and L2s were 36, 167, and 334, respectively (Figure 1A–C). Thus, the use of PD4251 allows for the removal of noise from CeHM in unwashed samples by selecting COPAS readings with a green fluorescence value above the background. On Day 5 post-L1 feeding (d5pL1f) of the parental population, the progeny were clearly separated from the adults using whole-body morphology parameters of ToF (time-of-flight, a measure of size) and EXT (extinction, a measure of optical density) (Figure 1D–F). 

**Figure 2:**
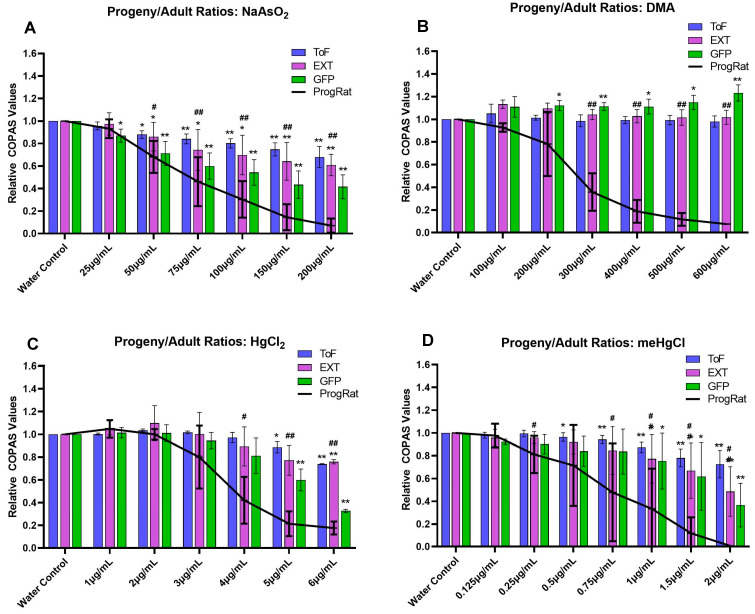
COPAS-measured progeny ratios and adult morphology parameters (**A**–**D**). Effects on parental TOF (blue bars), EXT (purple bars), green fluorescence (GFP, green bars), and progeny-to-adult ratios (black line) relative to matched (negative) water controls for sodium arsenite (NaAsO_2_), dimethylarsinic acid (DMA), mercury chloride (HgCl_2_), and methylmercury chloride (meHgCl). Data points and standard deviation error bars represent a minimum of 3 independent experiments. Student’s *t*-test *p*-values for parental morphology * (<0.05), ** (<0.005). Student’s *t*-test *p*-values for progeny to adult ratios # (<0.05), ## (<0.005).

For sodium arsenite (NaAsO_2_), the lowest observed effect levels (LOELs) for significant decreases in the progeny ratio as well as the parental ToF and EXT all occurred at 50 µg/mL (385 µM) (Figure 2A). Given that the GFP expression levels in PD4251 have previously been reported to remain steady with arsenic or mercury exposure [38], changes in green fluorescence were not an anticipated feature in the design of this progeny to adult population ratio assay. For NaAsO_2_, however, adult green fluorescence was the most sensitive measure of toxicity, with a LOEL of 25 µg/mL (192 µM), possibly reflecting increased levels of protein misfolding and/or degradation or changes in muscle mass and/or *myo-3* gene expression with arsenic exposure. The progeny ratio LOEL for dimethylarsinic acid (DMA) of 300 µg/mL (2200 µM) was much higher than for NaAsO_2_, while adult ToF and EXT were not affected by DMA at the tested concentrations (Figure 2B). Adult green fluorescence increased with increasing DMA. Gravid *C. elegans* were observed to thrash less with increasing DMA, although this was documented only as part of the routine microscopy notations and was not quantified. Therefore, the increased green fluorescence may reflect increased levels of GFP but could also be the result of altered posture or behavior during passage through the COPAS laser assessment chamber.

The progeny ratios for mercury chloride (HgCl_2_) decreased significantly at 4 µg/µL (15 µM), while LOELS for changes in adult ToF and green fluorescence were at 5 µg/mL (18 µM) HgCl_2_ (Figure 2C). The progeny ratio LOEL for methylmercury chloride (meHgCl) was 0.25 µg/mL (1 µM), although there was too much variability from among meHgCl experiments for the 0.5 µg/mL meHgCl mean progeny ratio decrease of 28% to reach statistical significance (Figure 2D). The LOEL for reductions in adult ToF was 0.5 µg/mL (2 µM) meHgCl, while both EXT and green fluorescence decreased at 1.0 µg/mL meHgCl.

### 3.2. Germline Health—Apoptosis 

**Figure 3:**
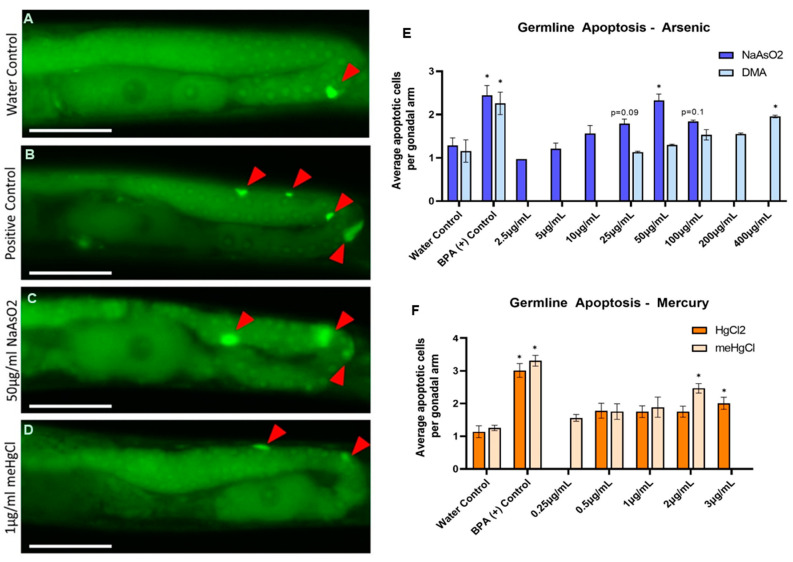
Assessment of germline apoptosis (**A**–**D**). Representative examples of acridine-orange-stained *C. elegans* following (**A**) water, (**B**) 100 µM BPA (positive control), (**C**) 50 µg/mL (385 µM) NaAsO_2_, and (**D**) 1 µg/mL meHgCl (4 µM) exposure. Images portray a focal plane in the posterior gonadal arm, with visible apoptotic nuclei. Red arrowheads identify apoptotic germ cell nuclei. Scale bar: 50 µm. (**E**,**F**) Number of apoptotic nuclei per gonadal arm of adult worms exposed to NaAsO_2_ and DMA (**E**) or HgCl_2_ and meHgCl (**F**) *n* = 3–4 repeats, 25–30 worms each. Student’s *t*-test *p*-values * (<0.05).

Germline apoptosis assessment measures the health of developing oocytes during the late prophase, when synapsis- and recombination-dependent checkpoint activation result in programmed germline nuclear cell death [34,39]. Acridine orange stains apoptotic cells [34], and these can be visualized in the *C. elegans* germline via fluorescence microscopy. *C. elegans* were exposed to water (negative control, Figure 3A), BPA (100 µM) (positive control, Figure 3B), or organic or inorganic arsenic or mercury for twenty-four hours post-mid-L4 stage, and were then stained with acridine orange to assess germline apoptosis. For NaAsO_2_ exposed worms, 50 µg/mL (385 µM) elicited a significant increase in germline apoptosis when compared to the control (1.22 apoptotic nuclei per gonadal arm ± 0.14 SEM vs. 2.33 ± 0.3 SEM, Figure 3E). Concentrations above 50 µg/mL did not kill the worms but did affect germline size (Figure 3C) and background fluorescence, making it difficult to clearly visualize the germline and count apoptotic nuclei. DMA significantly affected germline apoptosis only at the highest tested concentration of 400 µg/mL (2900 µM) (1.95 ± −0.04 SEM. Figure 3E). For both forms of mercury, evident germline morphology defects were present (Figure 3D) at all concentrations tested, and higher concentrations could not be tested as general degradation in the size and shape of the germline complicated the evaluation. Only the highest mercury concentrations tested, 3 µg/mL (11 µM) HgCl_2_ and 2 µg/mL (8 µM) meHgCl, elicited a significant increase in apoptotic germline nuclei when compared to the water control (2.01 ± 0.29 SEM, 2.47 ± 0.25 SEM respectively, Figure 3F).

### 3.3. Epigenetic Germline de-Silencing—Histone Regulation 

**Figure 4:**
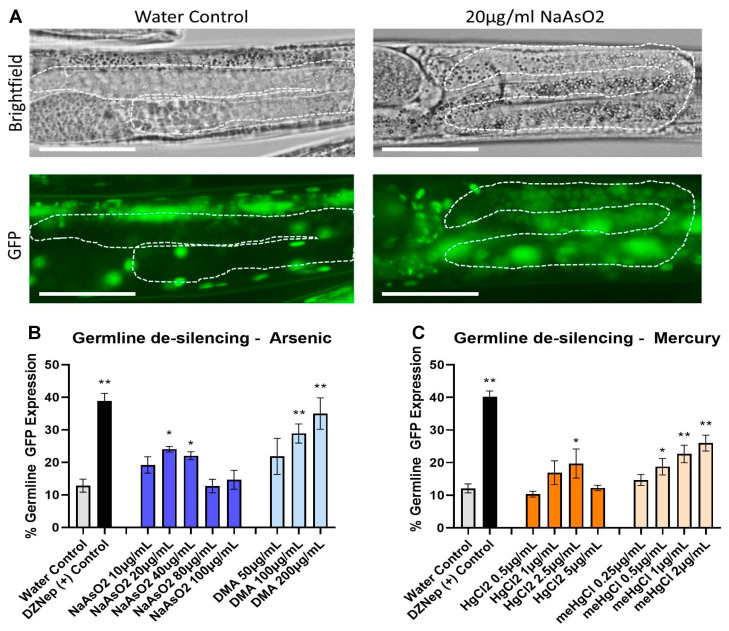
Assessment of germline histone regulation. (**A**) Brightfield and GFP images of silenced (negative) germline GFP (outlined in white) from a water control, and de-silenced (positive) germline GFP from a 20 µg/mL (155 µM) NaAsO_2_-exposed *C. elegans*. Scale bar: 50 µm. (**B**,**C**) Percentage of worms displaying germline de-silencing (y axis) at each exposure (x axis). *n* = 3–5, 30 worms each; Student’s *t*-test *p*-values * *p* ≤ 0.05, ** *p* ≤ 0.005.

In the healthy *C. elegans* germline, repetitive transgenes were regulated similarly to repetitive element silencing in mammalian germ cells [40,41]. The NL2507 *C. elegans* strain carries a repetitive GFP-tagged transgene that is expressed ubiquitously throughout the worm, with the exception of the germline where under normal conditions it is epigenetically silenced. This is carried out via a balance of repressive and activating histone modifications that keep repetitive transgenes from being expressed. The NL2507 strain can therefore be a useful reporter of the disruption to the histone modifications elicited by toxicant exposures via GFP de-silencing (or GFP expression) in the germline [35,42,43]. Utilizing this strain, germline epigenetic effects stemming from inorganic and organic arsenic and mercury exposure were assessed. *C. elegans* were exposed from the mid-L4 stage (50–52 h post-L1 feeding) for 24 h, encompassing the window from the L4 stage to Day 1 of adulthood (when gonadogenesis has been completed) [44]. Arsenic and mercury responses were compared to the water vehicle control, of which a low rate of de-silencing was observed (11.84 ± 1.19% SEM), and the DZnep (100 µM) positive control (39.72 ± 1.34% SEM) [35]. NaAsO_2_ at 20–40 µg/mL (155–310 µM) induced a significant de-silencing effect in the germline with a 22–24% increase from the control (24.05 ± 0.85%, 22.04 ± 1.12%, Figure 4A). The higher concentrations tested exhibited germline shrinkage (also observed in the germline health experiments), potentially disrupting and/or masking specific effects on the germline epigenome. At least 100 µg/mL (750 µM) of organic DMA was required to see a similar effect (28.8 ± 2.95%, Figure 4B) compared to NaAsO_2_, but there were no evident effects on germline morphology up to 200 µg/mL (1500 µM) DMA. In mercury-exposed worms, HgCl_2_ induced a significant de-silencing effect at 2.5 µg/mL (9 µM) (19.72 ± 4.46 SEM) but failed to do so at double the concentration (Figure 4C), likely due to increased gonadal shrinkage/dysmorphology at higher concentrations. Organic meHgCl induced a de-silencing germline effect at a 5-fold-lower concentration than the inorganic form (0.5 µg/mL (2 µM), 18.76 ± 2.52% SEM), and showed a dose–response of an increasing de-silencing effect through 2 µg/mL (12 µM). Starting at 1 µg/mL meHgCl and above, germlines were smaller and/or malformed, and worms appeared to be unhealthier overall. For these assays, worms were analyzed individually, and it was noted that as concentrations for each chemical increased, germline morphology was increasingly affected as well. If worms were stiff or not moving after the 24 h exposure, that concentration was not utilized as general toxicity may be affected more than the specific endpoint being investigated.

## 4. Discussion

The effects of arsenic and mercury can vary widely based on chemical form; therefore, safety assessments for one form do not always apply to other forms [45,46]. The FDA and EPA have set guidelines for inorganic arsenic in foods, and for mercury species in drinking water and seafood, but further studies are required for a better understanding of the effects of organic forms of arsenic [20,21,22,23,47,48]. Inorganic arsenic is a reproductive toxicant [49]; however, less is known about the reproductive effects of DMA [20]. This *C. elegans* study assessed progeny to adult ratios, germline apoptosis, and germline epigenetic regulation for DMA and NaAsO_2_, along with inorganic HgCl_2_ and organic meHgCl (Table 2), which have more thoroughly studied reproductive effects in mammalian models.

In studies using laboratory mammals, both organic and inorganic forms of mercury have demonstrated dose-dependent effects on fertility [50,51]. Organic methylmercury can elicit (or induce) effects on DART endpoints at lower concentrations than inorganic mercury due to its ability to cross the placenta and blood–brain barrier [52,53]. *C. elegans* is a simple organism that cannot model these mammalian systems, but it can model germ stem cell maintenance and differentiation, meiosis, reproductive output, and embryogenesis [15]. 

For inorganic arsenic, many studies in rodents have identified reductions in litter size only at exposures that were also maternally toxic [49,54,55]. Similarly for adult *C. elegans* exposed to inorganic arsenic, we found that the LOEL for a significant decrease in the ratio of progeny to adults of 50 µg/mL NaAsO_2_ (385 µM) was the same as the LOEL for reduced gravid adult body size and optical density (Figure 2A). In rodents, the effects on progeny after early exposure to inorganic arsenic (delayed incisor eruption, delayed bilateral eye opening, poor slant board performance) are seen at much lower concentrations than the maternal effects [54,55]. In a previous study, we found developmental delay and developmental hypoactivity at 10 µg/mL NaAsO_2_ [28], indicating that, as with laboratory mammals, developmental effects of inorganic arsenic exposure are seen in *C. elegans* at lower concentrations than the maternal effects. 

The *C. elegans* strain used in the progeny to adult ratio assay described here strongly expresses GFP via the *myo-3* promoter, and its expression level was previously shown to be unresponsive to concentrations of sodium arsenite or mercury that strongly activated genes in the stress response pathways [37,38]. GFP fluorescence is dependent on correct protein folding, and arsenic exposure results in protein misfolding [56,57]; therefore, the reduced green fluorescent signal in gravid adults exposed to 25 µg/mL or more NaAsO_2_ could be due to conserved arsenite-induced protein unfolding. However, arsenic exposure in humans is also associated with reduced muscle mass [58], suggesting that the reduced GFP could have been caused by changes in *C. elegans* muscle mass or *myo-3* gene expression. 

The progeny ratio LOEL for DMA was 300 µg/mL (2200 µM), a 6-fold concentration increase relative to the NaAsO_2_ LOEL (Figure 2B), but the same as the previously reported LOEL for hypoactivity in juvenile *C. elegans*, and only slightly higher than the *C. elegans* 200 µg/mL LOEL for developmental delay [28]. These similarly high LOEL concentrations across various endpoints in both juveniles and adults are consistent with DMA inducing systemic toxicity, rather than effects on specific biological processes. The body size and optical density of gravid adults were not altered at any tested concentration of DMA, but green fluorescence readings increased at concentrations of DMA ≥200 µg/mL (Figure 2B). This study did not assess motility, but decreased locomotion was noted via microscopy observation in gravid adults exposed to high concentrations of DMA. Additionally, in a separate study of *C. elegans* adults, the LOEL for hypoactivity with DMA was 300 µg/mL (manuscript in preparation). Therefore, increased green fluorescence could reflect changes in muscle cells, greater protein stability with DMA exposure relative to NaAsO_2_, or altered behavior or muscle tone during laser assessment.

Regarding mercury, HgCl_2_ significantly decreased progeny ratios at 4 µg/µL (15 µM), while the LOEL for meHgCl was much lower at 0.25 µg/mL (1 µM). There was a good deal of variability from one experiment to the next with both forms of mercury, resulting in large standard errors (Figure 2C,D). As with NaAsO_2_, significant reductions in progeny ratios were seen at mercury concentrations that also reduced parental body size and optical density. This is consistent with rodent studies investigating oral exposure to HgCl_2_, where LOEL effects for litter size and maternal maximum tolerable dose were similar [26,59,60]. In identified meHgCl oral exposure studies that also assessed HgCl_2_, when an effect on litter size was observed, it was seen at meHgCl concentrations lower than seen with HgCl_2_ (0.02–0.5 mg/kg/day meHgCl vs. 0.46–1.65 mg/kg/day HgCl_2_) [59,60,61].

To assess the reproductive-related effects of arsenic and mercury on the germline, exposures to inorganic and organic arsenic and mercury were conducted during the window in which *C. elegans* completed gonadogenesis and reached reproductive age [44]. The germline is established in early embryogenesis and is then maintained throughout development and in the adult gonad. We assessed germline apoptosis, an integral part of oogenesis that can be readily visualized in *C. elegans* as developing oocytes exit the pachytene stage of meiotic prophase [34]. NaAsO_2_ had a dose-dependent increase in germline apoptosis, with a LOEL of 50 µg/mL (385 µM); higher NaAsO_2_ concentrations altered gonadal morphology, making assessment difficult. At eight times the LOEL concentration of NaAsO_2_, DMA had a germline apoptosis LOEL of 400 µg/mL (2900 µM). For NaAsO_2_, germline apoptosis, progeny ratio, and parental morphology were statistically altered at the same concentration of 50 µg/mL, five times the concentration required for developmental delay and developmental hypoactivity in our previous study [28]. This is consistent with a recent study investigating the effects of NaAsO_2_ on mice, where the concentration that induced oocyte apoptosis was close to the maternally toxic concentration [62].

In contrast to arsenic, both assessed forms of mercury had LOELs at the highest concentrations that could be tested (2 µg/mL (11 µM) meHgCl, and 3 µg/mL (8 µM) HgCl_2_) before germline and overall morphology was affected. There are few rodent studies that directly assess the effects of oral mercury exposures on female germ cells, as most studies focus on male endpoints [51]. One study on female mice determined that although it is clear that methylmercury chloride is harmful to the female reproductive system, it is difficult to determine whether effects target oocytes or cause physiological damage to the mother [63]. This correlates with observing apoptosis at higher concentrations that also affect morphology. Germline assays, unlike progeny output, are more laborious as they require the direct assessment of one *C. elegans* germline at a time. As this is not a higher-throughput assessment, worms were only included if the germline remained visible, although morphology changes were evident. A more thorough investigation could compare germline size and apoptotic nuclei ratios, while here we propose that counts on healthier germline nuclei could indicate a specific effect as opposed to general toxicity.

We also evaluated the potential effects on the germline epigenome. In a previous gene expression assessment, our lab demonstrated that the direction of expression of genes involved in transcriptional regulation was toward the condensation of chromatin and the repression of transcription, potentially affecting biological processes including reproduction and transgenerational epigenetic regulation [28]. The previous assessment was conducted on whole worms at concentrations relevant to developmental delay. Here, we assessed the epigenetic effects on the developed *C. elegans* gonad, at a reproductive relevant stage. Histone modifications fluctuate during germ cell development, and thus play a key role in the establishment of chromatin environment and gametogenesis [64]. Studies in mice assessing oocytes and aging found that changes in histone acetylation and methylation can result in oocyte dysfunction and infertility [65,66]. The distribution and regulation of chromatin marks are well characterized in *C. elegans* [67,68,69] and repetitive transgenes are silenced in the germline via repressive histone modifications in a similar manner to the silencing of repetitive elements in mammalian germ cells [42,70]. Rodent and *C. elegans* assays have demonstrated epigenetic alterations, including to histone modifications, directly induced by NaAsO_2_ and meHgCl exposures, as well as in their offspring [71,72,73,74,75,76]. Multi-generational studies on zebrafish and mice have demonstrated the long-term effects of mercury exposure passed down for multiple generations, indicating the potential effects on the epigenome [77,78,79]. However, most of these studies were not specific to reproductive endpoints and did not compare different forms of arsenic and/or mercury. Our work seeks to fill this gap and demonstrate how *C. elegans* can be potentially employed as a model for the more rapid assessment of effects on the germline epigenome. 

We evaluated a disruption in histone regulation with the use of an epigenetic reporter strain [42]. The histone methyltransferase inhibitor and potential epigenetic therapeutic drug DZNep was used as the positive control [28,80,81,82]. NaAsO_2_ disrupted germline histone regulation at a LOEL of 20 µg/mL (155 µM), while inorganic DMA had a LOEL of 100 µg/mL (750 µM) for this endpoint. Compared to the progeny ratio and germline apoptosis, the germline epigenome assay is more sensitive for arsenic, suggesting potential arsenic specific effects on the epigenome. In mercury-exposed worms, inorganic HgCl_2_ disrupted germline histone regulation significantly at a LOEL concentration of 2.5 µg/mL (9 µM), while the organic meHgCl LOEL was at a 5-times-lower concentration than the inorganic form (0.5 ug/mL (2 µM)). For developmental delay and germline histone regulation, the LOELs were similar for both forms of mercury tested. 

For the histone regulation assay, the worms were individually evaluated, and although this is a quicker method than in vivo mammalian studies, it is also a laborious experiment that shows high sensitivity. The transgenic strain that was utilized has been available for over two decades, and the field could be much improved with upgraded transgenics, allowing for a truly higher-throughput assay making use of a biosorter, as was the case for the progeny output assays. 

This work contributes to the understanding of accuracy and fit-for-purpose categories for *C. elegans* toxicity screening; however, much larger panels of chemicals with known effects on mammals need to be tested in order to move toward using this model for regulatory purposes.

## 5. Conclusions

This study demonstrates the importance of assessing multiple endpoints to identify specific effects at lower concentrations that do not induce general toxicity. Overall, *C. elegans* results for exposures to mercury and inorganic arsenic reflect what is known from mammalian oral exposures, while less-studied DMA was far less toxic for the reproductive-related endpoints assessed here. Germline histone regulation was altered at arsenic and mercury concentrations that also induced developmental delay in juvenile *C. elegans*. For arsenic, but not mercury, germline histone regulation was altered at concentrations below those that affected the progeny/adult ratios. For these endpoints, organic and inorganic forms of arsenic and mercury were ranked meHgCl > HgCl_2_ > NaAsO_2_ > DMA. Where comparable mammalian oral toxicity data were available, concordant effects suggest that *C. elegans* data have the potential to complement existing in vivo studies. *C. elegans* data can be a useful component in integrated assessments that include other NAMs and small model organisms, potentially contributing to weight of evidence assessments, hazard ID, and regulatory decision making.

## Figures and Tables

**Table 1:** Test Chemicals.

Test Chemical	Abbreviation	CAS RN	Molecular Weight
Sodium (meta)arsenite	NaAsO_2_	7784–46-5	129.91
Dimethylarsinic acid (DMA^V^)	DMA	75–60-5	138.00
Mercury(ii) chloride	HgCl_2_	7487–94-7	271.50
Methylmercury chloride	meHgCl	115–09-3	251.08

**Table 2:** Oral exposure LOEL summary for the *C. elegans* reproductive-toxicity-related endpoints in this study.

Chemical	Assay	*C. elegans* Strain	Stage (Duration) of Exposure	LOEL µg/mL (µM)
Sodium (meta)arsenite	Progeny ratio	PD4251	yA (2 days)	50 (385)
	Germline apoptosis	N2 wild type	L4 (24 h)	50 (385)
	Germline histone regulation	NL2507	L4 (24 h)	20 (155)
Dimethylarsinic acid (DMA^V^)	Progeny ratio	PD4251	yA (2 days)	300 (2200)
	Germline apoptosis	N2 wild type	L4 (24 h)	400 (2900)
	Germline histone regulation	NL2507	L4 (24 h)	100 (750)
Mercury(ii) chloride	Progeny ratio	PD4251	yA (2 days)	4 (15)
	Germline apoptosis	N2 wild type	L4 (24 h)	3 (11)
	Germline histone regulation	NL2507	L4 (24 h)	2.5 (9)
Methylmercury chloride	Progeny ratio	PD4251	yA (2 days)	0.25 (1)
	Germline apoptosis	N2 wild type	L4 (24 h)	2 (8)
	Germline histone regulation	NL2507	L4 (24 h)	0.5 (2)

## Data Availability

Data are contained within the article.
